# The emergency to home project: impact of an emergency department care coordinator on hospital admission and emergency department utilization among seniors

**DOI:** 10.1186/1865-1380-7-18

**Published:** 2014-05-07

**Authors:** Christopher Matthew Bond, Elizabeth A Freiheit, Lesley Podruzny, Alianu Akawakun Kingsly, Dongmei Wang, Jamie Davenport, Abram Gutscher, Cathy Askin, Allison Taylor, Vivian Lee, Queenie Choo, Eddy Samuel Lang

**Affiliations:** 1 Department of Emergency Medicine, Foothills Medical Centre, Room C231, 1403-29 St NW, Calgary, Alberta T2N 2 T9, Canada; 2 Department of Family Medicine, University of Calgary, 2500 University Drive NW, Calgary, Alberta T2N 1 N4, Canada; 3 Alberta Health Services, Seventh Street Plaza, 10030-107 St NW, Edmonton, Alberta T5J 3E4, Canada; 4 Alberta Health Services, Reporting Services, Data Integration Measurement and Reporting, NWII, Suite 300, 4520-16 Ave NW, Calgary, Alberta T3B 0 M6, Canada

**Keywords:** Geriatric EM care, ED aftercare, Home care, Recidivism, Seniors, Elderly, Falls, Discharge planning

## Abstract

**Background:**

Seniors comprise 14% to 21% of all emergency department (ED) visits, yet are disproportionately larger users of ED and inpatient resources. ED care coordinators (EDCCs) target seniors at risk for functional decline and connect them to home care and other community services in hopes of avoiding hospitalization.

The goal of this study was to measure the association between the presence of EDCCs and admission rates for seniors aged ≥ 65. Secondary outcomes included length of stay, recidivism at 30 days, and revisit resulting in admission at 30 days.

**Methods:**

This was a matched pairs study using administrative data from eight EDs in six Alberta cities. Four of these hospitals were intervention sites, in which patients were seen by an EDCC, while the other four sites had no EDCC presence. All seniors aged ≥ 65 with a discharge diagnosis of fall or musculoskeletal pathology were included. Cases were matched by CTAS category, age, gender, mode of arrival, and home living environment. McNemar’s test for matched pairs was used to compare admission and recidivism rates at EDCC and non-EDCC hospitals. A paired *t*-test was used to compare length of stay between groups.

**Results:**

There were no statistically significant differences for baseline admission rate, revisit rate at 30 days, and readmission rate at 30 days between EDCC and non-EDCC patients.

**Conclusions:**

This study showed no reduction in senior patients’ admission rates, recidivism at 30 days, or hospital length of stay when comparing seniors seen by an EDCC with those not seen by an EDCC.

## Background

Compared with younger persons, older adults use emergency services at a higher rate, are more likely to be admitted or have repeat emergency department (ED) visits, and experience higher rates of adverse health outcomes after discharge [1]. In addition to their increased patterns of ED use, frail elderly patients seek care for falls, a presenting complaint not often seen in the young [1]. Furthermore, injuries in the elderly often require more intensive therapy and use disproportionately more resources than would similar injuries in young patients [2]. Several systematic reviews have examined the impact of interventions targeted at improving outcomes for seniors visiting the ED, with mixed results [3,4]. The nature of these interventions has ranged from comprehensive geriatric assessments (CGA) with specially trained ED teams, to telephone follow-up of seniors discharged from the ED [5-7]. Desired outcomes have been variable, and have included quality of life improvement, slowing progression of functional decline, and reducing ED visits and recidivism [3,4]. One randomized Australian study showed that seniors’ admission rates at 30 days could be reduced through assessment by a CGA team prior to their initial ED discharge [8]. Another study showed that a nurse discharge plan coordinator dedicated specifically to the discharge planning care of elderly patients, reduced the proportion of unscheduled ED return visits and facilitated the transition from ED back home and into the community health care network [9].

In order to address the complex needs of seniors visiting the ED, Alberta Health Services^a^ (AHS), the provincial health authority, commissioned the Emergency to Home Project (E2H) in 2009. E2H connects seniors visiting the ED with services in the community through the efforts of the Emergency Department Care Coordinator (EDCC). The EDCC is a specialized nursing role dedicated to supporting seniors and their caregivers to return home safely after an ED visit. EDCCs educate and advocate for patients and their families by identifying seniors needs outside the ED through conducting an assessment and providing referrals to home care and other community-based services.

In some cases, referral may also involve admission of the patient to an inpatient transition bed until community resources become available. The referrals and supports provided by EDCCs are intended to facilitate meeting seniors’ health care needs in the community, thereby reducing repeat ED visits, hospitalization, and hospital length of stay (LOS). Selection of seniors for EDCC consultation was based on the following factors. Seniors who were pre-existing home care clients in the community automatically triggered an EDCC consult when they registered in the ED. If no pre-existing home care was in place, a physician or nurse-initiated consult to the EDCC was required.

The goal of this study was to determine whether the presence of EDCCs would decrease the admission rate of seniors aged ≥ 65 who have an ED discharge diagnosis of fall or other musculoskeletal (MSK) pathology. We hypothesized that seniors aged ≥ 65 with falls and MSK injuries are frequently admitted to hospital because of their lack of social supports and the risks associated with ED discharge.

## Methods

### Ethics review

The research question and methods were evaluated using the Alberta Research Ethics Community Consensus Initiative (ARRECI) tool [10]. ARRECI is a four-step, web-based ethics screening tool designed by Alberta Innovates–Health Solutions (formerly the Alberta Heritage Foundation for Medical Research). The project’s primary purpose, details of data collected, and risk to participants are inputted into the tool, which then risk stratifies the research project and suggests whether Research Ethics Board approval is needed. Using this tool, it was established that no formal ethics board review was necessary, as the study was low risk and used only anonymous data.

### Study design and data sources

This study used a matched pairs study design using administrative data from eight EDs in six cities in Alberta, Canada. The Data Integration, Measurement and Reporting (DIMR), and Information Management and Technology Services (IMTS) departments of AHS provided the data. Nosologists from IMTS are responsible for coding and abstracting patient diagnoses based on ICD-10 codes and record disposition and LOS data using common tracking systems. Separate information analysts from DIMR subsequently report this data, along with demographic and other information. Seniors presenting with falls and MSK complaints were targeted as they represent a group that is more likely to benefit from ED interventions that arrange a safe discharge plan. The primary outcome was hospital admission rate on index visit. Secondary outcomes included LOS, revisit rate at 30 days, and admission rate at 30 days. In addition to looking at all seniors aged ≥ 65, cohorts of seniors with identical MSK discharge diagnoses were also analyzed.

### Interventions

**Figure 1:**
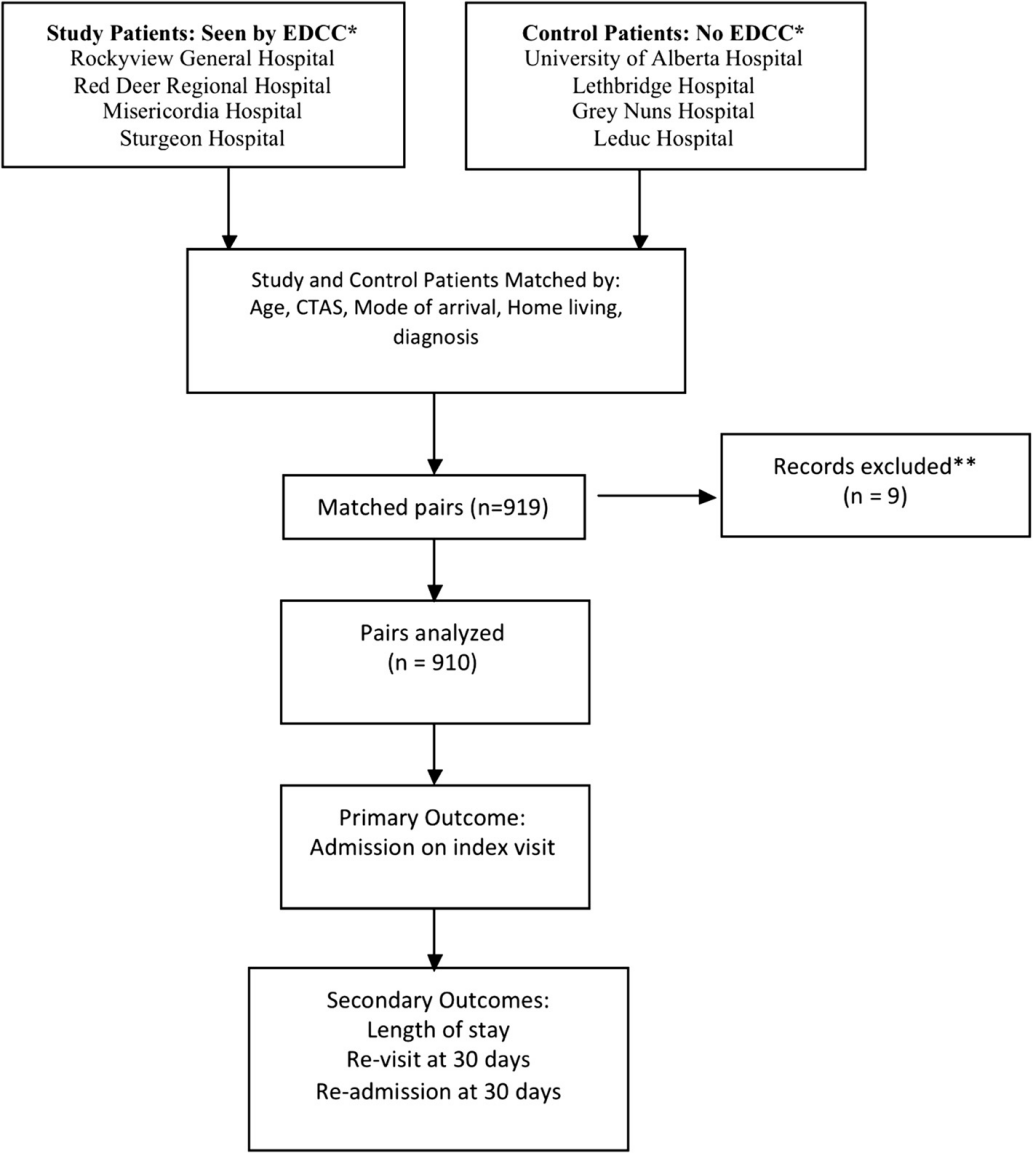
**Study flow.** *Data Sources: E2H Clerical Staff and DIMR (Data Integration, Measurement and Reporting). **Exclusions: Missing outcome data or left ED without being seen.

Four of the eight hospitals were intervention sites, in which patients were seen by an EDCC, while the remaining four sites were used as a comparator group and had no EDCC presence during the study period of April 1, 2010 to March 31, 2011. The four intervention sites were chosen from EDs with EDCC resources in place, funded by the AHS E2H project. The intervention and control hospitals were selected based on comparable characteristics: annual number of ED visits and patient acuity according to the Canadian Triage and Acuity Scale (CTAS) [11]. Figure 1 shows the study flow diagram and lists specific hospital sites. Interventions at the study hospitals were variable, and are detailed in Table 1.

**Table 1:** E2H pilot project sites and main project activities

**Site**	**Main project activities**
Misericordia Community Hospital	Introduced new EDCC role
Sturgeon Community Hospital	Introduced new EDCC role
Red Deer Regional Hospital	Increased EDCC hours by providing an additional 1.4 EDCC full time equivalents for evening and weekend coverage, introduced clerical support for the EDCC, expanded home care coverage, and enhanced the role of the ED pharmacist and physiotherapist.
Rockyview General Hospital	Introduced clerical support for Transition Coordinators. The EDCC role was already established at this site; increased clerical support was needed to support the volume of referrals.

### Study population

Seniors aged ≥ 65 with an ICD-10 discharge diagnosis of fall, fracture, sprain, strain, laceration, contusion, superficial injury, or bursitis were included for possible analysis. Patients with a discharge diagnosis of hip fracture or trimalleolar ankle fracture were excluded due to their 100% need for operative intervention. Patients who had presented to an Alberta ED for a MSK complaint within the previous 30 days were also excluded.

Overall, 919 patients seen by the EDCC during their ED visit were matched with patients who had not been seen by the EDCC. Of this group, 9 pairs were excluded from the study: 2 pairs were excluded because outcome data was not available for one member of each pair and 7 pairs were excluded because one member of each pair left the emergency room before assessment could be completed. The analysis dataset consisted of 910 matched pairs.

In the analysis dataset, most pairs (832) were matched on age (plus or minus 3 years), CTAS, mode of arrival (ambulance or walk-in), and living arrangements (home or institution) (Table 2). Of these, 231 pairs were also matched on diagnosis type (patients were matched by above criteria, but also had identical discharge diagnosis, e.g., Colles fracture, rib contusion); 45 pairs were matched on age, CTAS, and mode of arrival only; 25 pairs were matched on only age and CTAS; and 8 pairs were not CTAS matched, but differed by one. As all matches were made from like-hospitals, all pairs were also matched on hospital type. The criteria used to match pairs are shown in Table 2. Of the 910 pairs, 188 (20.7%) were matched on age, CTAS, mode of arrival, and living arrangements and had identical MSK diagnoses; 44 pairs (4.8%) were matched on age, CTAS, mode of arrival, living arrangements, and had the diagnosis of “tendency to fall – not elsewhere classified (NEC)”. Matching was performed by a trained data abstractor who was blinded to the outcome data.

**Table 2:** Matched pairs by matching criteria

**# Matched pairs (n = 910)**	**Age (±3 yr)**	**CTAS score**	**Mode of arrival**^**1**^	**Living arrangements**^**1**^	**Discharge diagnosis**	**Hospital type**
601	X	X	X	X		X
231	X	X	X	X	X	X
45	X	X	X			X
25	X	X				
8	X		X	X		X

^1^AHS is the single health authority responsible for delivering health care in the province of Alberta, Canada.

### Analysis

A matched analysis was performed on the study sample, using McNemar’s test for dichotomous outcomes (admission at baseline, revisit within 30 days, and admission within 30 days after baseline). A paired *t*-test was used to compare LOS between EDCC patients and controls. The analysis was performed on the entire study population and then on subgroups of patients with identical MSK diagnoses and “tendency to fall – NEC” diagnoses. The identical MSK diagnoses subgroup compared patients with the exact same discharge diagnosis, in an effort to control for comparison of MSK injuries of potentially differing severity. The tendency to fall NEC group represented patients who did not have an MSK diagnosis, but an underlying co-morbidity (e.g., urinary tract infection, exacerbation of chronic disease) that led to their ED presentation.

The data was provided by DIMR in Microsoft Excel (2007) files and then imported to Stata Software: Release 12 for statistical analysis [12].

## Results

Baseline characteristics of the study population are shown in Table 3.

**Table 3:** Baseline characteristics

	**Non-EDCC patients, n = 910**	**EDCC patients, n = 910**
Age, years (Std. dev)	80.5 (8.0)	80.5 (8.0)
Female, number (%)	623 (68.5)	643 (70.7)
CTAS = 2, number (%)	69 (7.6)	69 (7.6)
CTAS = 3	575 (63.2)	578 (63.5)
CTAS = 4	257 (28.2)	253 (27.8)
CTAS = 5	9 (1.0)	10 (1.1)
Arrival by ground ambulance, number (%)	518 (56.9)	541 (59.5)
Lives in institution, number (%)	147 (16.2)	194 (21.3)

Of the 1,820 patients included in the study (EDDC and non-EDCC), 547 (30.1%) were admitted to hospital at the baseline and 354 (19.5%) revisited emergency during the 30-day follow-up period; 140 patients (7.7%) were admitted to hospital within the follow-up period.

Our study was designed with 90% power to detect a difference of 6% between discordant pairs, assuming 989 pairs and approximately 20% discordant pairs [13]. However, our sample of 910 pairs had 15.6% in EDCC admit/non-EDCC non-admit, and 13.7% EDCC non-admit/non-EDCC admit, a difference of only 1.9%. As a result, our sample of 910 had only 18.5% power to detect this small 1.9% difference. However, a 1.9% difference, while statistically significant, may not be clinically significant.

Comparison of baseline admission rates found no statistically significant differences between EDCC patients and non-EDCC patients in the odds of baseline admission, revisit at 30 days or readmission at 30 days:

•Admission rates (OR = 0.88; 95% CI, 0.69 to 1.12)

•Revisit rates at 30 days (OR = 1.19; 95% CI, 0.95 to 1.51)

•Readmission rates at 30 days (OR = 1.03; 95% CI, 0.73 to 1.46).

LOS data on initial hospital admission was available for 93 patients where both EDCC patients and controls were admitted. Mean LOS for EDCC patients was 8.15 days longer than mean LOS for controls (*P* = 0.065), a non-significant difference. Looking only at those with MSK diagnoses where both EDCC patients and controls were admitted (n = 14), mean LOS for EDCC patients was 6.2 days shorter than controls (*P* = 0.34). Looking only at those with “tendency to fall – NEC” diagnoses where both EDCC patients and controls were admitted (n = 20), mean LOS for EDCC patients was 7.4 days shorter than controls (*P* = 0.33). None of these differences are statistically significant.

Table 4 shows dichotomous outcomes for the entire sample. Tables 5 and 6 show subgroup analysis where diagnoses are MSK and “tendency to fall – NEC”. Results are similar to the whole group analyses.

**Table 4:** Dichotomous outcomes: whole sample

	**Frequency of outcome number, (%) n = 1820**	**Number of EDDC patients with outcome (%) n = 910**	**Number of non-EDCC patients with outcome (%) n = 910**	**Number (%) of pairs where EDCC experienced the outcome and non-EDCC did not, n = 910**	**Number (%) of pairs where non-EDCC experienced the outcome and EDCC did not, n = 910**	**McNemar’s test: p-value**
Admissions	547 (30.1)	282 (31.0)	265 (29.1)	142 (15.6)	125 (13.7)	0.3275
Revisits	354 (19.5)	164 (18.0)	190 (20.9)	132 (14.5)	158 (17.4)	0.1420
Readmission	140 (7.7)	69 (7.6)	71 (7.8)	63 (6.9)	65 (7.1)	0.9296

**Table 5:** Dichotomous outcomes: patients with primary MSK diagnosis

	**Frequency of outcome number, (%) n = 376**	**Number of EDCC patients with outcome (%) n = 188**	**Number of non-EDCC patients with outcome (%) n = 188**	**Number (%) of pairs where EDCC experienced the outcome and non-EDCC did not, n = 188**	**Number (%) of pairs where non-EDCC experienced the outcome and EDCC did not, n = 188**	**McNemar’s test: p-value**
Admissions	84 (22.3)	48 (25.5)	36 (19.1)	25 (13.3)	13 (6.9)	0.0730
Revisits	76 (20.2)	34 (18.1)	42 (22.3)	25 (13.3)	33 (17.6)	0.3581
Readmission	28 (7.4)	13 (6.9)	15 (8.0)	12 (6.4)	14 (7.5)	0.8450

**Table 6:** Dichotomous outcomes: patients with primary diagnosis of “tendency to fall”

	**Frequency of outcome number, (%) n = 88**	**Number of EDCC patients with outcome (%) n = 44**	**Number of non-EDCC patients with outcome (%) n = 44**	**Number (%) of pairs where EDCC experienced the outcome and non-EDCC did not, n = 44**	**Number (%) of pairs where non-EDCC experienced the outcome and EDCC did not, n = 44**	**McNemar’s test: p-value**
Admissions	68 (77.3)	36 (81.2)	32 (72.3)	12 (27.3)	8 (18.2)	0.5034
Revisits	12 (13.6)	6 (13.6)	6 (13.6)	5 (11.4)	5 (11.4)	1.000
Readmission	6 (6.8)	4 (9.1)	2 (4.6)	3 (6.8)	1 (2.3)	0.6250

## Discussion

This retrospective matched study examined the impact of an ED-based intervention for geriatric patients in the form of assessment by an EDCC. This study found no statistically significant differences in the hospital admission rates, LOS or ED re-visits within 30 days of an initial visit for seniors seen by an EDCC and those not seen by an EDCC.

There are several possible explanations for the outcomes of patients included in this study. Selection bias in the sample of EDCC-assessed seniors used in the study may have affected the admission rate on the index visit. Seniors visiting an ED are not randomly selected for assessment by the EDCC; rather, the EDCC likely identifies seniors at risk of falling in the community who would be otherwise discharged from the ED. By bringing these patients to the attention of the ED staff and having them admitted, this may offset some of the admissions the EDCCs are themselves preventing. Further, many of the patients being seen by the EDCC are frail and present with multiple co-morbidities. Many of these patients may have been admitted regardless of EDCC involvement. Unfortunately, because of the retrospective study design, screening tools that could potentially identify patients at higher risk of readmission were not used for patient selection [14].

An important goal in the care of seniors is to deliver targeted multidisciplinary care to achieve improved health outcomes [8]. However, the impact of ED-based interventions in reducing elderly hospitalization and recidivism has been variable [4]. In the DEED II study, comprehensive geriatric assessment of ED patients aged 75 and older showed a lower rate of hospital admissions at 30 days and a reduction in ED admissions at 18 months [8]. The 30 day re-visit and re-hospitalization rates in DEED II were 22% and 16%, respectively, compared with 19.5% and 7.7% in our study [8]. However, the DEED II study population differed in that all patients assessed were discharged from the ED on their initial visit, and followed-up with a CGA. Thus, it is difficult to compare this with the patients in our study. Furthermore, our study targeted seniors with MSK diagnoses, and thus we may expect a smaller impact than that seen in DEED II. CGA for discharged ED patients was also performed in a study by Mion et al., in this case seniors aged 65 and older were assessed by an advance practice nurse and referred to appropriate outpatient services [15]. This study showed a reduction in nursing home admissions at 30 days, but no reductions in repeat ED visits or hospitalizations. Guttman et al., evaluated the impact of a nurse discharge plan coordinator in a Canadian setting, a role similar to an EDCC, on unscheduled seniors ED revisits at 8 and 14 days [9]. Although a modest positive effect was shown in reducing unscheduled revisits, again this study population consisted exclusively of patients that were discharged from the ED. In a study by Gagnon et al., nurse case management of elderly patients discharge from the ED resulted in higher rates of ED readmission, with no improvement in other outcomes [16]. In all of these studies, seniors were discharged from the ED on the index visit, and all enrolled patients were community-dwelling and not institutionalized.

In our study, all seniors aged ≥ 65 could potentially be seen and interviewed by the EDCC, including many in whom hospital admission would likely be unavoidable. However, some seniors were not seen by the EDCC due to extremes of acuity or already supportive living situations. For example, a trauma patient requiring intensive care unit admission or an ill medical patient would not be candidates for EDCC intervention, as there was no possibility of imminent discharge. Similarly, a patient with minor complaints and excellent home supports (e.g., children and spouse providing care) would not be seen by the EDCC. Therefore, a sensitivity analysis could not be performed to compare patients seen by the EDCC who were not eligible for EDCC intervention. Based on the baseline characteristics of the participants, the study patients were of moderate to high acuity, with 70% being CTAS 2 or 3, and 57% to 60% arriving by ambulance. The average age of 80.5 ± 8.0 and large percentage of institutionalized patients also indicates this study’s patients were of higher than average risk. While no statistically significant outcomes were identified in this study, several trends were noted. Whole group revisits at 30 days trended toward a reduction for patients who were seen by an EDCC when compared with the no EDCC group (*P* = 0.14). In the subgroup of patients with identical MSK diagnosis, there was a trend toward increased admission on index visit in the study group, when compared with the controls (*P* = 0.07). Finally, in the LOS evaluation, the mean LOS for the EDCC patients was 8.15 days longer than controls with a trend toward statistical significance (*P* = 0.065).

## Conclusions

This study showed no reduction in baseline admission rate, revisits at 30 days, readmissions at 30 days, or hospital LOS for seniors aged ≥ 65 seen by an EDCC, when compared with similar seniors not seen by an EDCC. However, this study had several limitations that may have affected the results, including the study design, sample bias, and variable nature of interventions at different hospital sites. There may be additional benefits of EDCC intervention, such as improved patient satisfaction, quality of life, and a reduction in functional decline. In the future, we would suggest further evaluation of other benefits provided by the EDCC role, as well as longer-term evaluation of in-patient admission and ED utilization rates at 6 months or 1 year.

Potential limitations of this study were numerous, and included study design, heterogeneity of interventions, and chosen outcomes. First, a retrospective matched study design is inferior to a prospective, randomized study design. Without a prospective design, patient selection and screening is not possible, and from previous studies of geriatric interventions, selection of an appropriate intervention population is paramount [4]. Our population was heterogeneous in both the type and severity of their discharge diagnoses, and while representative of a typical ED population, some of these patients may not be appropriate for EDCC intervention. Second, the nature of the EDCC intervention varied among hospital sites, in that the program was implemented from scratch at two sites and expanded upon at the other two sites. Third, it has been difficult to show the positive impact of an ED intervention on the outcomes chosen in this study, as has been documented in multiple previous studies [4]. Other outcomes, such as reduction in functional decline and quality of life improvement, may be more appropriate proxies for quality improvement in this domain. Fourth, the administrative database did not record patient function (e.g., activities of daily living), mobility, and co-morbidities. Matching using these additional criteria would strengthen the overall conclusions and their absence had potential to confound the results. Fifth, the positive downstream effects of the EDCC on recidivism may not be apparent using a 30 day cutoff. Perhaps a window of 6 months or longer is more appropriate for analysis of these outcomes.

## Endnote

^a^AHS is the single health authority responsible for delivering health care in the province of Alberta, Canada.

## Abbreviations

AHS: Alberta Health Services; CGA: Comprehensive geriatric assessments; CTAS: Canadian Triage and Acuity Scale; DIMR: Data Integration, Measurement and Reporting; ED: Emergency department; EDCC: Emergency Department Care Coordinator; E2H: Emergency to Home Project; LOS: Length of stay; MSK: Musculoskeletal; NEC: Not elsewhere classified.

## Competing interests

The authors declare that they have no competing interests.

## Authors’ contributions

EAF, KAA, DW, and VL performed statistical analysis of the data. CA, JD, VL, and AT gathered the data, performed data linkage and created the dashboards for analysis. AT, JD, and QC were responsible for the Emergency to Home Project leadership and oversight. CMB, EL, AT, JD, VL, and QC conceived the study design. CMB drafted the manuscript, all other authors contributed to revisions for the final submission. All authors read and approved the final manuscript.
